# Effects of dietary salt restriction on home blood pressure in diabetic patients with excessive salt intake: a pilot study

**DOI:** 10.3164/jcbn.19-61

**Published:** 2019-10-08

**Authors:** Emi Ushigome, Chikako Oyabu, Keiko Iwai, Nobuko Kitagawa, Aya Kitae, Tomonori Kimura, Isao Yokota, Hidetaka Ushigome, Masahide Hamaguchi, Mai Asano, Masahiro Yamazaki, Michiaki Fukui

**Affiliations:** 1 Department of Endocrinology and Metabolism, Kyoto Prefectural University of Medicine, Graduate School of Medical Science, 465 Kajii-cho, Kawaramachi-Hirokoji, Kamigyo-ku, Kyoto 602-8566, Japan; 2 Department of Endocrinology and Metabolism, Kyoto First Red Cross Hospital, 749 Honmachi 15-chome, Higashiyama-ku, Kyoto 605-0981, Japan; 3 Department of Biostatistics, Graduate School of Medicine, Hokkaido University, Kita 8, Nishi 5, Kita-ku, Sapporo, Hokkaido 060-0808, Japan; 4 Department of Organ Transplantation and General Surgery, Kyoto Prefectural University of Medicine, Graduate School of Medical Science, 465 Kajii-cho, Kawaramachi-Hirokoji, Kamigyo-ku, Kyoto 602-8566, Japan

**Keywords:** dietary salt restriction guidance, home blood pressure, intervention study, telemedicine system, type 2 diabetes mellitus

## Abstract

The aim of the present study was to examine whether dietary salt restriction guidance is beneficial for dietary salt restriction and lowering of home blood pressure in patients with diabetes with excessive salt intake. We performed an intervention trial of 37 people with type 2 diabetes and excessive salt intake. National registered dietitians provided dietary salt restriction guidance to each patient at the start of the study. All participants were instructed to perform triplicate morning and evening home blood pressure measurements using home blood pressure telemonitoring system. Daily salt intake at 2 months and 6 months was significantly lower than that at baseline; the difference was 0.8 [95% confidence interval (CI): 0.2–1.4, *p* = 0.009] g and 0.7 (95% CI: 0.1–1.3, *p* = 0.009) g, respectively. Morning systolic blood pressure at 2 months and 6 months was significantly lower than that at baseline; the difference was 2.7 (95% CI: 0.2–5.1, *p* = 0.034) mmHg and 5.8 (95% CI: 0.5–11.1, *p* = 0.034) mmHg, respectively. This intervention study revealed, for the first time, that dietary salt restriction guidance provided by a national registered dietitian is beneficial for reducing daily salt intake and home blood pressure in people with diabetes with excessive salt intake.

## Introduction

Through observational studies it has been shown that most people consume an excess amount of sodium and that excessive salt intake is one of the most important etiological factors for hypertension and may cause various diseases, such as stroke, coronary heart disease, and nephropathy.^(1)^ Evidence on the hypotensive effects and reduction in the risk of cardiovascular diseases with dietary salt restriction has accumulated.^(2)^ Therefore, in many guidelines on the management of hypertension, salt restriction is recommended as a lifestyle modification.

Both diabetes and hypertension are important risk factors for cardiovascular disease. When the two diseases coexist, the incidence of cardiovascular disease markedly increases.^(3)^ Therefore, strict control of blood pressure (BP), as well as blood glucose, is important for the prevention and treatment of microvascular and macrovascular diseases in people with diabetes and hypertension. For BP control, the self-measurement of BP at home (HBP) has been reported to be a more reliable predictor of prognosis compared with clinic BP.^(4–6)^ Thus, the utility and the priority of HBP measurement have been widely accepted.

The objective of this study was to clarify whether dietary salt restriction guidance provided by a national registered dietitian is beneficial for reducing salt intake and HBP in people with diabetes with excessive salt intake (equal to or more than 6 g per day in people with hypertension and equal to or more than 7 g per day in female or 8 g per day in male people without hypertension).

## Material and Methods

### Participants

We sequentially recruited 57 people with type 2 diabetes who had regularly attended the diabetes outpatient clinic at the Hospital of the Kyoto Prefectural University of Medicine from December 2014 to July 2016. Inclusion criteria were as follows: 40–80 years old, type 2 diabetes mellitus, not taking sodium-glucose transporter 2 (SGLT-2) inhibitors, salt intake equal to or more than 6 g per day in people with hypertension^(7)^ and salt intake equal to or more than 7 g per day in women and 8 g per day in men without hypertension.^(8)^ Hypertension was defined as clinic BP over 130/80 mmHg and/or HBP over 125/75 mmHg pressure even after lifestyle modifications, which corresponds to the criteria for hypertension in the Japanese Society of Hypertension Guidelines 2014.^(7)^ Hypertension was also defined as the current use of antihypertensive drugs. No BP-level criterion was used for study inclusion. Exclusion criteria were as follows: secondary hypertension or malignant hypertension, history of myocardial infarction, cerebrovascular disease or hospitalization for angina pectoris within 6 months prior to inclusion, advanced renal dysfunction (serum creatinine equal to or more than 2.0 mg/dl or current treatment by dialysis), changing antihypertensive medication and/or antidiabetic medication within 1 month prior to inclusion, atrial fibrillation or severe arrhythmia, life-threatening condition like malignant tumor, or judged by a supervising physician to be unsuitable as a study patient. The diagnosis of type 2 diabetes was based on the American Diabetes Association criteria.^(9)^

### Study design

This was an intervention trial to examine the impact of dietary salt restriction guidance on daily salt intake and HBP using an HBP telemonitoring system in people with diabetes with excessive salt intake. We could not determine the sample size before the study because no previous reports had described the relationship between dietary salt restriction guidance provided by a national registered dietitian and HBP.

All participants monitored their HBP during the study. National registered dietitians delivered dietary salt restriction guidance (asking about what the patient usually eats, offering a new food plan to achieve a reduced sodium diet, suggesting products and foods, and explaining how to prepare them so that they are tasty) for about 30 min to each patient once at the start of the study. We calculated daily salt intake and HBP before (at baseline), 2 months, and 6 months, after the guidance.

All procedures were approved by the local Research Ethics Committee and were conducted in accordance with the Declaration of Helsinki, and with informed consent obtained from all participants (RBMR-E-349-2).

### HBP measurements

We followed the methods of Ushigome *et al.*^(10)^. In briefly, HBP was self-measured using an automated BP monitor, HEM-7251G (Omron Healthcare Co., Ltd., Kyoto, Japan). All participants were instructed to perform triplicate morning and evening BP measurements with at least 1 min between recordings at least 5 days per week during the study. They were instructed to perform the morning measurements of BP within 1 h of awakening, before eating breakfast or taking any drugs, with the patient seated and rested for at least 5 min, and perform the evening measurements of BP in a similar fashion just before going to bed (eating was prohibited for over 1 h before measurements).^(11)^ The cuff was placed around the non-dominant arm and the position of the cuff was maintained at the level of the heart. Proper cuff size was determined based on arm circumference. The standard arm cuff and tube were used for HBP measurements in all participants. The HBP readings were visible to study participants. Participants need not have kept a diary to record the measured values as the BP device was capable of transmitting date, time, and measurement results automatically and immediately after each measurement via mobile phone line to the server of a BP management system, Medical LINK^®^ (Omron Healthcare). This provided reliable data collection for physicians. We calculated the mean of 3 measurements per morning and 3 per evening each day, and then the level of HBP was computed from those 14 days at baseline, 2 months, and 6 months after the dietary salt restriction guidance for each individual in this study.

### Data Collection

We also followed the methods of our previous report.^(10)^ Blood samples were taken in the morning for biochemical measurements at the time of study entry. Hemoglobin A1c, serum lipid profile, and other biochemical data were determined using standard laboratory measurements. Daily salt intake was estimated by spot urine sample. Estimated daily salt intake was calculated from the following equation: 0.0585 × 21.98 × {urinary sodium/urinary creatinine × (14.89 × body weight (kg) + 16.14 × height (cm) – 2.04 × age – 2,244.45)}^0.392^.^(12)^ Urinary albumin excretion (UAE) was measured using an immunoturbidimetric assay. The average value for UAE was determined from triplicate urine collections. Hemoglobin A1c was expressed as National Glycohemoglobin Standardization Program units. Each patient’s data, including age, duration of diabetes, smoking and alcohol consumption status (assessed by an interview), and antihypertensive medication were gathered at the time of study entry. Retinopathy was assessed from chart reviews. Nephropathy was graded into 3 stages depending on UAE as follows: normoalbuminuria, UAE less than 30 mg/g Cr; microalbuminuria, 30–300 mg/g Cr; or macroalbuminuria, more than 300 mg/g Cr. Neuropathy was defined as the diagnostic criteria for diabetic polyneuropathy proposed by the Diabetic Neuropathy Study Group.^(13)^ Macrovascular complication was defined as the presence of previous cardiovascular disease, cerebrovascular disease, or arteriosclerosis obliterans based on the clinical history or physical examination.

### Statistical analysis

Baseline characteristics were reported as means with SD or numbers. Paired *t* tests were used to compare daily salt intake and HBP at baseline, 2 and 6 months after the dietary salt restriction guidance. The difference in HBP with 95% confidence interval (CI) was also presented.

We performed the subgroup analyses according to age (equal to or more than 70 years old and less than 70 years old) and use of antihypertensive drugs (the presence and absence of antihypertensive medication) because age-related salt sensitivity and antihypertensive drugs may affect HBP.

We used SPSS statistical package, ver. 19.0J (SPSS, Inc., Chicago, IL) for analyses. All analyses were two-sided, and *p*<0.05 was considered statistically significant.

## Results

**Fig. 1:**
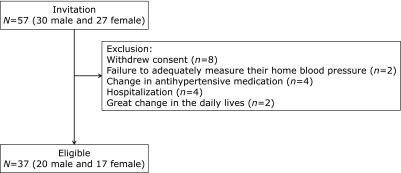
Participants’ flowchart.

Of the 57 people who met the inclusion criteria for assessments, 20 were excluded from the study analysis, primarily due to withdrawal of consent for the following reasons: refusal of dietary salt restriction guidance; reluctance to transmit each measurement results automatically to the BP management system; difficulty with self-measuring HBP, failure to adequately measure HBP due to hospitalization, a change in antihypertensive medication, and great change in their daily lives because of their partners’ hospitalization (Fig. 1). Consequently, 37 people comprised the study population (20 male and 17 female). Table 1 shows the baseline characteristics of the participants at study entry. The mean ± SD age and hemoglobin A1c were 67.7 ± 9.6 years and 7.0 ± 0.6%, respectively. Table 2 shows daily salt intake and HBP at baseline, 2 and 6 months after the dietary salt restriction guidance, as well as the difference in each parameter between baseline and after the guidance (baseline vs 2 months and baseline vs 6 months) with a 95% CI. Daily salt intake at 2 and 6 months was significantly lower than that at baseline; the difference was 0.8 (95% CI: 0.2–1.4) g and 0.7 (95% CI: 0.1–1.3) g, respectively. Morning systolic BP at 2 and 6 months was significantly lower than at baseline; the difference was 2.7 (95% CI: 0.2 to 5.1) mmHg and 5.8 (95% CI: 0.5 to 11.1) mmHg, respectively. Morning diastolic BP at 6 months was significantly lower than that at baseline; the difference was 4.4 (95% CI: 0.9–6.1) mmHg. Evening systolic BP at 2 and 6 months tended to be lower than at baseline; the difference was 3.1 (95% CI: −0.3–6.4) mmHg and 3.5 (95% CI: −0.5–6.8) mmHg, respectively. There were no significant differences in the mean evening diastolic BP during the study.

In people of 70 years of age or older, daily salt intake at 2 months was significantly lower while that at 6 months tended to be lower than that at baseline; the difference was 0.6 (95% CI: 0.0–1.2) g and 0.7 (95% CI: −0.1–1.7) g, respectively. Morning systolic BP at 2 and 6 months tended to be lower than that at baseline; the differencewas 2.0 (95% CI: −0.8–5.7) mmHg and 6.7 (95% CI: −1.5–14.9) mmHg, respectively. In people less than 70 years of age, daily salt intake at 2 and 6 months tended to be lower than that at baseline; the difference was 1.0 (95% CI: −0.2–2.1) g and 0.6 (95% CI: −0.3–1.6) g, respectively. Morning systolic BP at 2 and 6 months tended to be lower than that at baseline; the difference was 2.9 (95% CI: −1.3–7.2) mmHg and 5.4 (95% CI: −1.4–10.0) mmHg, respectively (Table 3).

In people receiving antihypertensive medication, there were no significant differences in daily salt intake during the study. Morning systolic BP at 2 and 6 months tended to be lower than that at baseline; the difference was 3.2 (95% CI: −0.1–6.4) mmHg and 6.4 (95% CI: −2.7–13.4) mmHg, respectively. In people without antihypertensive medication, daily salt intake at 2 and 6 months was significantly lower than that at baseline; the difference was 1.3 (95% CI: 0.6–2.0) g and 1.4 (95% CI: 0.2–2.5) g, respectively. Morning systolic BP at 6 months tended to be lower than that at baseline; the difference was 5.5 (95% CI: −0.2–13.1) mmHg (Table 4).

## Discussion

This intervention study revealed that the dietary salt restriction guidance provided by a national registered dietitian might be beneficial for reducing daily salt intake and HBP in people with diabetes with excessive salt intake.

An elevated BP increases the risks of cardiovascular diseases, renal dysfunction, and retinopathy in people with diabetes.^(7)^ In addition, hypertension is strongly and independently associated with diabetes because insulin resistance and hyperglycemia stimulate renal sodium absorption, which plays a key role in the pathogenesis of hypertension in people with diabetes.^(14,15)^ Current guidelines, which recommend salt restriction, are based on observational and interventional studies that show an association between excessive salt intake and increased BP, as well as the hypotensive effects of salt restriction.^(1,2,16)^ The mean salt intake in Japan is high. Therefore, salt restriction is essential for the prevention and treatment of hypertension and for the prevention of cardiovascular diseases. In contrast, for BP control, HBP has been reported to be a more reliable predictor of prognosis than clinic BP.^(5,17)^

In the present study, daily salt intake was significantly reduced by 0.8 g at 2 months and 0.7 g at 6 months after dietary salt restriction guidance provided by a national registered dietitian. Moreover, morning systolic BP was significantly reduced by 2.7 mmHg at 2 months and 5.8 mmHg at 6 months after dietary salt restriction guidance provided by a national registered dietitian. The Dietary Approaches to Stop Hypertension (DASH) trial studied the effect on BP of different levels of dietary salt in conjunction with the DASH diet, which is rich in vegetables, fruits, and low-fat dairy products, and showed that restriction of salt intake lowered BP.^(16)^ Reducing the salt intake from a high (8.38 g) to an intermediate (6.35 g) level reduced the systolic BP by 2.1 mmHg (*p*<0.001) with the control diet and by 1.3 mmHg (*p* = 0.03) with the DASH diet. A meta-analysis of randomized salt restriction trials concluded that a reduction of 100 mmol/day (6 g) in salt intake predicted a fall in BP of 7.11/3.88 mmHg (*p*<0.001 for both systolic and diastolic) in people with hypertension and 3.57/1.66 mmHg in normotensive individuals (systolic: *p*<0.001; diastolic: *p*<0.05). Furthermore, a modest restriction in salt intake for 4 or more weeks had a significant and important effect on BP in both hypertensive and normotensive individuals.^(18)^ In our study, the effect of the restriction in daily sodium intake and HBP at 6 months was confirmed. A meta-analysis of randomized controlled trials also suggests that moderate dietary salt restriction significantly reduces BP and proteinuria, proportionally to BP decline, in patients with chronic kidney disease.^(19)^

The increase in BP in response to salt intake differs according to age.^(20)^ Therefore, we performed age-specific analyses. There was no age-specific difference in the reduction of daily salt intake and HBP after the dietary salt restriction guidance in the present study.

The study population included people with hypertension receiving antihypertensive medication (*n* = 22, 59%). Because antihypertensive drugs may affect HBP, we performed subgroup analyses according to the presence or absence of antihypertensive medication. The effect of the dietary salt restriction guidance on lowering daily salt intake in people who were not receiving antihypertensive medication was larger than that in those receiving antihypertensive medication. In contrast, the effect of the guidance on the lowering of morning systolic BP in people receiving antihypertensive medication was larger than that in those not receiving antihypertensive medication. The difference in daily salt intake and HBP at baseline between the 2 groups might affect these results.

The strengths of the present study include that we used the HBP telemonitoring system, which transmitted measurement results automatically and immediately to the server of a BP management system. This automatically provided reliable aggregated data rather than trusting poorly-entered patient logbooks.^(21)^ Furthermore, HBP measurements were gathered for 14 days (a relatively long consecutive period).

We acknowledge that our study has some limitations. First, small sample sizes limited the statistical power. However, we could detect a statistically significant difference in daily salt intake and HBP before and after the dietary salt restriction guidance. Secondly, we had no data on the nutritional composition of the participants’ diet, except for salt intake. Intake of potassium, calcium, magnesium, dietary fiber, and protein might also affect HBP to the same extent as salt.^(16)^ Third, the sodium sensitivity of BP is reported to be enhanced in people with diabetes and/or metabolic syndrome, and it might affect our results. However, we do not have data on sodium sensitivity. Fourth, daily salt intake was estimated by the spot urine sample in this study. However, values obtained by the spot urine method correlated highly with those obtained by 24 h urinary sodium excretion,^(22)^ and usefulness of the spot urine method has been confirmed in population studies^(23)^ and in hypertensive patients.^(24)^ Fifth, this study design was not double-blind; therefore, some inherent bias cannot be completely ruled out. In the future, a randomized controlled trial of a larger sample sizes or a meta-analysis concerning this population is necessary. Finally, our study was unable to determine whether the restriction of daily salt intake and HBP would have led directly to better outcomes. Further studies are needed to test this hypothesis. Finally, it is not clear in the present study which is more important, nutritional guidance itself or the provision of nutritional guidance by a dietitian. We plan to conduct an intervention study to examine whether dietary salt restriction guidance provided by clinicians could also be beneficial for reducing daily salt intake and HBP in people with diabetes with excessive salt intake.

In summary, dietary salt restriction guidance provided by a national registered dietitian is beneficial for reducing daily salt intake and HBP in people with diabetes with excessive salt intake. Our results might contribute additive information for clinicians who are involved in the management of people with diabetes with excessive salt intake.

## Author Contributions

EU designed the study protocol, contributed to the collection of research data, performed data analyses, reviewed/edited the manuscript. CO also designed the study protocol, contributed to the collection of research data, performed data analyses, and reviewed the manuscript. KI, NK, AK, TK, HU, MH, MA and MY designed the study protocol, reviewed data reports, contributed to discussion, and reviewed the study manuscript. IY supervised data analysis, contributed to manuscript preparation, contributed to discussion, and reviewed the manuscript. MF designed the protocol, performed data analyses, drafted the manuscript, and was the principal investigator of the Graduate School of Medical Science, Kyoto Prefectural University of Medicine. All authors reviewed and provided edits and comments on manuscript drafts. EU is the guarantor of this work and, as such, had full access to all the data in the study and takes responsibility for the integrity of the data and the accuracy of the data analysis.

## Figures and Tables

**Table 1:** Baseline characteristics of the study participants

Characteristic	
*n* (male/female)	37 (20/17)
Age (years)	67.7 ± 9.6
Duration of diabetes (years)	14.2 ± 7.5
Body mass index (kg/m^2^)	23.3 ± 2.8
Hemoglobin A1c [% (mmol/mol)]	7.0 ± 0.6
	(53.0 ± 9.0)
Total cholesterol (mmol/L)	4.6 ± 0.7
Triglycerides (mmol/L)	1.4 ± 0.8
Creatinine (mg/dl)	0.8 ± 0.2
eGFR (ml/min/1.73 m^2^)	76.9 ± 27.4
Daily salt intake (g)	10.4 ± 2.2
Urinary sodium/potassium ratio	3.5 ± 1.8
Smoking status (current/past/never)	3/13/21
Alcohol consumption status (everyday/social/never)	8/8/21
Nephropathy (normo-/micro-/macroalbuminuria)	24/12/1
Retinopathy (NDR/SDR/PDR)	28/6/3
Neuropathy (–/+)	27/10
Macrovascular complication (–/+)	32/5
Hypoglycemic treatment (diet/OHA/insulin)	5/32/6
Antihypertensive medication (–/+)	15/22
Morning systolic blood pressure (mmHg)	135.2 ± 13.2
Morning diastolic blood pressure (mmHg)	78.0 ± 9.9
Evening systolic blood pressure (mmHg)	130.3 ± 12.6
Evening diastolic blood pressure (mmHg)	73.9 ± 10.3

Data are means ± SD or number. eGFR, estimated glomerular filtration rate; NDR, no diabetic retinopathy; SDR, simple diabetic retinopathy; PDR, proliferative diabetic retinopathy; OHA, oral hypoglycemic agent.

**Table 2:** Home blood pressure and daily salt intake before (baseline), 2 months, and 6 months after dietary salt restriction guidance

Characteristic	Baseline	2 months	6 months	Difference (95% CI) *p* value	
				(baseline vs 2 months)	(baseline vs 6 months)
Daily salt intake (g)	10.4 ± 2.2	9.6 ± 1.7	9.7 ± 2.0	0.8 (0.2–1.4) *p* = 0.009	0.7 (0.1–1.3) *p* = 0.025
Morning systolic blood pressure (mmHg)	135.2 ± 13.2	132.5 ± 13.4	129.4 ± 13.1	2.7 (0.2–5.1) *p* = 0.034	5.8 (0.5–11.1) *p* = 0.034
Morning diastolic blood pressure (mmHg)	78.0 ± 9.9	78.0 ± 11.0	73.6 ± 7.6	0.0 (−3.1–3.2) *p* = 0.986	4.4 (0.9–6.1) *p* = 0.009
Evening systolic blood pressure (mmHg)	130.3 ± 12.6	127.2 ± 16.2	126.8 ± 11.6	3.1 (−0.3–6.4) *p* = 0.074	3.5 (−0.5–6.8) *p* = 0.085
Evening diastolic blood pressure (mmHg)	73.9 ± 10.3	72.7 ± 9.7	72.5 ± 9.3	1.2 (−0.7–3.1) *p* = 0.212	1.4 (−1.2–3.4) *p* = 0.334

Data are means ± SD; HBP, home blood pressure; CI, confidence interval.

**Table 3:** Home blood pressure and daily salt intake before (baseline), 2 months, and 6 months after dietary salt restriction guidance in people 70 years old or over and less than 70 years old

Characteristic	Baseline	2 months	6 months	Difference (95% CI) *p* value	
				(baseline vs 2 months)	(baseline vs 6 months)
≥70 years old (*n* = 20)					
Daily salt intake (g)	10.3 ± 1.8	9.7 ± 1.8	9.6 ± 1.9	0.6 (0.0–1.2) *p* = 0.035	0.7 (−0.1–1.7) *p* = 0.082
Morning systolic blood pressure (mmHg)	139.1 ± 14.5	137.1 ± 13.4	132.3 ± 14.3	2.0 (−0.8–5.7) *p* = 0.127	6.7 (−1.5–14.9) *p* = 0.104
<70 years old (*n* = 17)					
Daily salt intake (g)	10.5 ± 2.6	9.5 ± 1.6	9.9 ± 2.2	1.0 (−0.2–2.1) *p* = 0.085	0.6 (−0.3–1.6) *p* = 0.180
Morning systolic blood pressure (mmHg)	130.1 ± 9.7	127.1 ± 11.5	124.6 ± 9.8	2.9 (−1.3–7.2) *p* = 0.158	5.4 (−1.4–10.0) *p* = 0.125

Data are means ± SD.

**Table 4:** Home blood pressure and daily salt intake before (baseline), 2 months, and 6 months, after dietary salt restriction guidance in people receiving and not receiving antihypertensive medication

Characteristic	Baseline	2 months	6 months	Difference (95% CI) *p* value	
				(baseline vs 2 months)	(baseline vs 6 months)
With antihypertensive medication (*n* = 22)					
Daily salt intake (g)	10.1 ± 2.7	9.6 ± 2.0	9.8 ± 2.1	0.5 (−0.4–1.3) *p* = 0.273	0.3 (−0.4–1.0) *p* = 0.426
Morning systolic blood pressure (mmHg)	138.7 ± 13.6	135.5 ± 11.9	132.3 ± 15.1	3.2 (−0.1–6.4) *p* = 0.053	6.4 (−2.7–13.4) *p* = 0.176
Without antihypertensive medication (*n* = 15)					
Daily salt intake (g)	10.9 ± 1.2	9.7 ± 1.1	9.6 ± 1.9	1.3 (0.6–2.0) *p* = 0.002	1.4 (0.2–2.5) *p* = 0.023
Morning systolic blood pressure (mmHg)	130.1 ± 11.2	128.1 ± 14.6	124.6 ± 7.5	1.9 (−2.3–6.2) *p* = 0.351	5.5 (−0.2–13.1) *p* = 0.057

Data are means ± SD.

## Abbreviations

BP: blood pressure
CI: confidence interval
DASH: Dietary Approaches to Stop Hypertension
HBP: self-measurement of BP at home
SGLT-2: sodium-glucose transporter 2
UAE: urinary albumin excretion
